# Technical note: unsafe rectal temperature measurements due to delayed warming of the thermocouple by using a condom. An issue concerning the estimation of the postmortem interval by using Henßge’s nomogram

**DOI:** 10.1007/s00414-015-1186-2

**Published:** 2015-05-14

**Authors:** Tristan Krap, Joris Meurs, Janine Boertjes, Wilma Duijst

**Affiliations:** Ars Cogniscendi Centre for Legal and Forensic Medicine, Wezep, Netherlands; Department of Anatomy, Embryology and Physiology, Academic Medical Centre, Amsterdam, The Netherlands; Van Hall Larenstein, Forensic Science, University of Applied Sciences, Postbus 1528, 8901 BV, Leeuwarden, Netherlands; Public Health Service, Department of Forensic Medicine, IJsselland, Netherlands

**Keywords:** Postmortem, Interval, Cooling, Model, Temperature, Nomogram

## Abstract

In some cases, in the Netherlands, an additional layer is being added to the thermocouple, used to measure the rectal temperature in medicolegal death investigations. Because of this deviation from the standard method, questions arose regarding the accuracy and precision of the measured temperature. Therefore, a cooling experiment was carried out on a round body made of agar with an average thermal conductivity of 0.454 W/(m °C) while measuring the temperature with and without an additional layer around the thermocouple for three different starting temperatures: 36, 30, and 27 °C. The results show a significant difference between the measured values for the first 5 min when comparing with and without the additional layer. Further, a decrease in precision is present within the first minutes when using an additional layer. Therefore, it is concluded that it is best to measure the rectal temperature without an additional layer around the thermocouple and caution should be taken when measuring with an additional layer.

## Introduction

In the Netherlands, the forensic medical examiner performs a necropsy at the crime scene and does little more in the mortuary. The next stop in the medicolegal investigation is the forensic pathologist who performs the autopsy. The rectal temperature is, in most cases, measured at the scene of the crime to estimate the postmortem interval by using Henßge’s nomogram, supported by the degree and state of livor and rigor mortis. National guidelines from the Dutch Forensic Medical Society, in use by the departments of Forensic Medicine of the Public Health Service, state that a protective layer should be added to the thermocouple for rectal measurements. Arguments for this deviation from the standard method as suggested by Henßge are as follows [8]:

- To prevent contamination
- For ease of insertion
- For hygienic purposes.

In practice, the layers used to follow the guidelines are a nitrile glove or a condom. Further, the guidelines state that the temperature should be measured after several minutes and if possible after 1 h in situ.

Theoretically, adding layers results in an obstruction in the quasi-equilibrium that the thermodynamic system wishes to achieve; it has an insulating effect. Thermal energy can be transferred by radiation, conduction, and convection. In the situation of measuring the rectal temperature, the thermal energy is transferred by conduction by making direct contact with the surrounding tissues, and to a lesser extend due to radiation from these tissues and convection. According to Fourier’s law, heat is transferred from more energetic particles to less energetic particles in its environment without physical transportation of these particles [7]. Each layer between the high energetic particles and the less energetic particles is an obstruction of the transference of energy. The impact of the obstruction on the flow of energy is determined by its thermal conductivity, expressed as λ. Adding a layer results in an obstruction of the quasi-equilibrium. The factor suffering most from these additional layers is time, and in time, the entire body will transport its remaining thermal energy to its surroundings. Since time and temperature are the dependents used for a PMI estimation based on the nomogram of Henßge, it is important to obtain a reliable measurement. According to Hubig et al., errors in input variables of the nomogram of Henßge, which seem to be insignificant, can lead to wrong postmortem interval estimates [9]. Since the nomogram of Henßge is primarily used as tool for estimating the postmortem interval, measurement errors should be brought to a minimum.

The effect of the additional layer was investigated by measuring the temperature of a body made of agar with and without the additional layer simultaneously for a duration of 90 min. Additionally, the difference between a nitrile glove and a condom as an additional layer was studied.

## Materials and methodology

### Research equipment

- † Thermometer_environment_: Beurer HM16, resolution 0.1 °C and accuracy 0.1 °C
- † 2 • Thermometer_ball_: Testo 108, resolution 0.1 °C and accuracy 0.5 °C; thermocouple type T
- † Thermostat_water bath_: Julabo P4 basic
- † Balance: Mettler Toledo PG12001-s
- † Water bath (0.8 m • 0.4 m • 0.4 m)
- † Spherical mold, internal diameter 0.2 m, and a volume of 4.19 × 10^−3^ m^3^
- † Condoms (latex)
- † Nitrile gloves (acrylonitrile butadiene rubber, NBR)
- † SPSS 22 for Mac
- † Microsoft^®^ Excel^®^ for Mac

### Research materials

- † Technical agar, no.3, Oxoid LP0013, ordered January 2014, stored cool and dry
- † Purified water
- † Petroleum jelly

### Concentration of agar

**Fig. 1:**
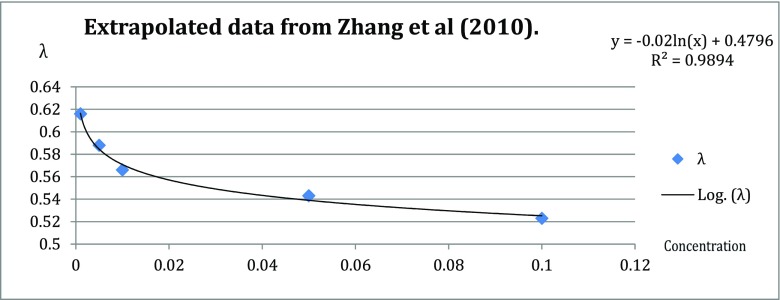
Extrapolated data from Zhang et al., Table 3 page 863. The logarithmic function of the trend line (correlation coefficient 0.99) is determined by using Microsoft^®^ Excel^®^ 2008 for Mac v. 12.3.6; the function reads *y* = −0.02*ln*(*x*) + 0.4796 [13]

To investigate the effect of the additional layers surrounding the thermocouple, a body was created of technical agar, with a λ of 0.454 W/(m °C) (see Table 1 for several tissues and their associated λ). The requirements for the ball were stability and enough cohesion, and sufficient, but not too much, rigidity to prevent it from rupturing during the experiment. The thermal conductivity of 0.454 W/(m °C) falls within the range of human skeletal muscle at a temperature of 37 °C and produced a ball that met the requirements. The chosen λ of 0.454 W/(m °C) was converted to a concentration of 3.6 % *w*/*v* (weight (g) / volume (ml) × 100). The concentration was calculated by using the data from Zhang et al., who investigated the thermal conductivity of several concentrations agar at different temperatures. Figure 1 shows the extrapolated logarithmic function based on several concentrations at 20 °C. The logarithmic function is *y* = −0.02*ln*(*x*) + 0.4796, where *y* = λ and *x* = concentration % *w*/*v* [2, 13].

**Table 1:** Different types of tissue with their associated thermal conductivity measured at a specific temperature.

Tissue type:	λ (W/(m °C))	Temperature (°C)	Reference
Sheep resting skeletal muscle	0.478	21	[1]
Human skeletal muscle	0.449–0.546	37	[2]
Human subcutaneous adipose	0.200–0.246	37	[2]
Human colon	0.556 ± 0.009	37	[2]
Human cardiac muscle	0.492–0.562	37	[2]

$$ 0.454=-0.02 ln(3.6)+0.4796 $$

### Agar gel preparation and creation of the agar body and subsequent experiments

To obtain an agar concentration of 3.6 % *w*/*v*, ∼1 l of purified water was added to 36 g of technical agar [10]. Each ball contained 3.1 l of agar solution. To improve the dissolution of the agar in purified water, the water was heated to its boiling point.

The agar solution was poured into a mold, a hollow spherical ball made of rubber added with a thin layer of petroleum jelly for the ease of removal of the rubber mold. In the mold, two elongated cylindrical objects where placed with a diameter of 0.004 m and a length of 0.08 m, on opposite sites, to insert the thermocouples without causing the solidified agar to rupture. The agar solution had a higher temperature than desired; because of this, the ball was cooled by placing it in a bucket with ice prior to removing the mold. To stabilize the agar ball during the experiment, one fourth of the bottom part was cut off transversally, resulting in an average weight of 3 kg. The agar ball was then brought to the desired temperature by using a warm water bath, heated by a Julabo thermostat and verified with a Testo 108 thermometer. The temperatures used in the experiments are 36 °C (*N* = 10 for both additional layers), 30 °C (*N* = 5 for both additional layers), and 27 °C (*N* = 5 for both additional layers). The ball was dried by using paper towels and two thermocouples were inserted, one with an additional layer and one without. The environment in which the experiments took place is best described as a laboratory with closed windows, occasionally minor movement of air due to employees walking around. The experiments were carried out away from the windows to avoid interference from sun radiation. For each experiment, a new ball was created following the abovementioned protocol.

**Fig. 2:**
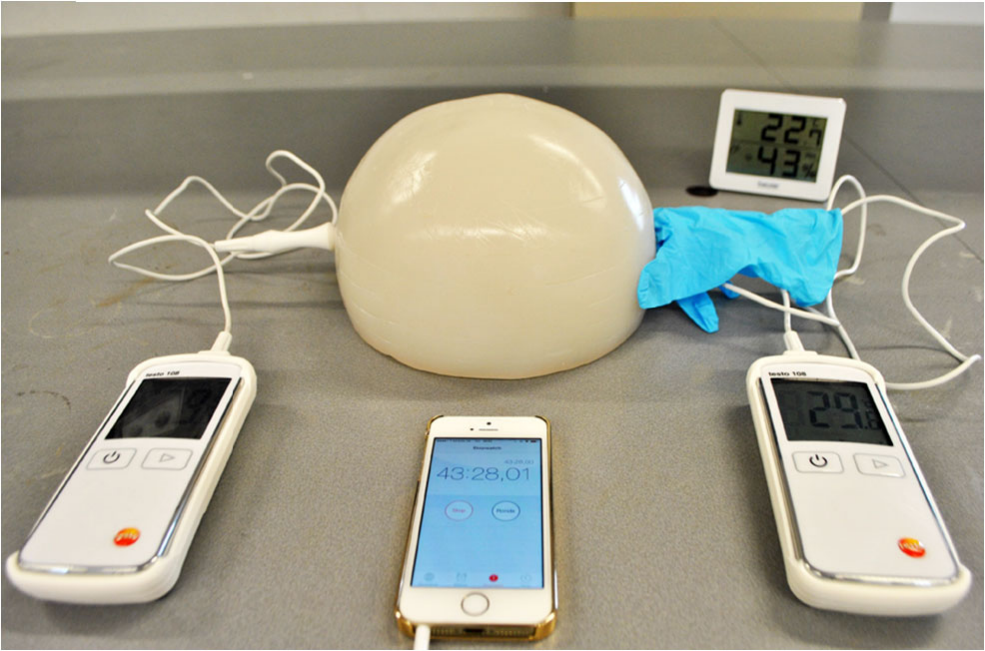
The setup of the experiment. A ball of agar with a λ of 0.454 W/(m °C) has reached a temperature of 29.8 °C after 43″ and 28′; the environmental temperature is 22.7 °C. The right thermocouple has an additional layer around it, in this case, a nitrile glove. Photographic copyright belongs to J. Boertjes

For 90 min, the two temperatures of the ball and the environmental temperature were logged manually after 17, 33, and 50 s and at intervals of 30 s for the first 5 min, 1 min for the first 10 min, and 5 min for the remainder of the 90 min. Figure 2 shows the setup of the experiment. The overall average ambient temperature was 22.4 °C ± 2 °C (95.4 % CI).

### Statistical analysis

The acquired data from each experiment was analyzed by using SPSS 22 for Mac, and the 2σ confidence interval (CI) for each temperature plotted and the significance was determined by applying an independent Student’s *t* test to the following groups:

- Without condom versus with condom
- Without nitrile glove versus with nitrile glove

In order to make a valid comparison between the measurements taken with a condom to those taken with a nitrile glove, it was necessary to determine whether both cooling experiments cooled down in the same manner. Therefore, a Student’s *t* test was first applied to the measurements taken without the additional layer, the blank, and subsequently on the ambient temperatures during both experiments. Student’s *t* tests were applied on the following groups in the following order:

- Without condom versus without nitrile glove
- Ambient temperature condom versus ambient temperature nitrile glove
- And finally, with condom versus with nitrile glove

## Results

The ambient temperatures during all the experiments, at all time points, did not significantly differ from each other (*ρ* > 0.05), and the temperature measurements without an additional layer did not significantly differ from each other either (*ρ* > 0.05).

### Starting temperature 36 °C

**Fig. 3:**
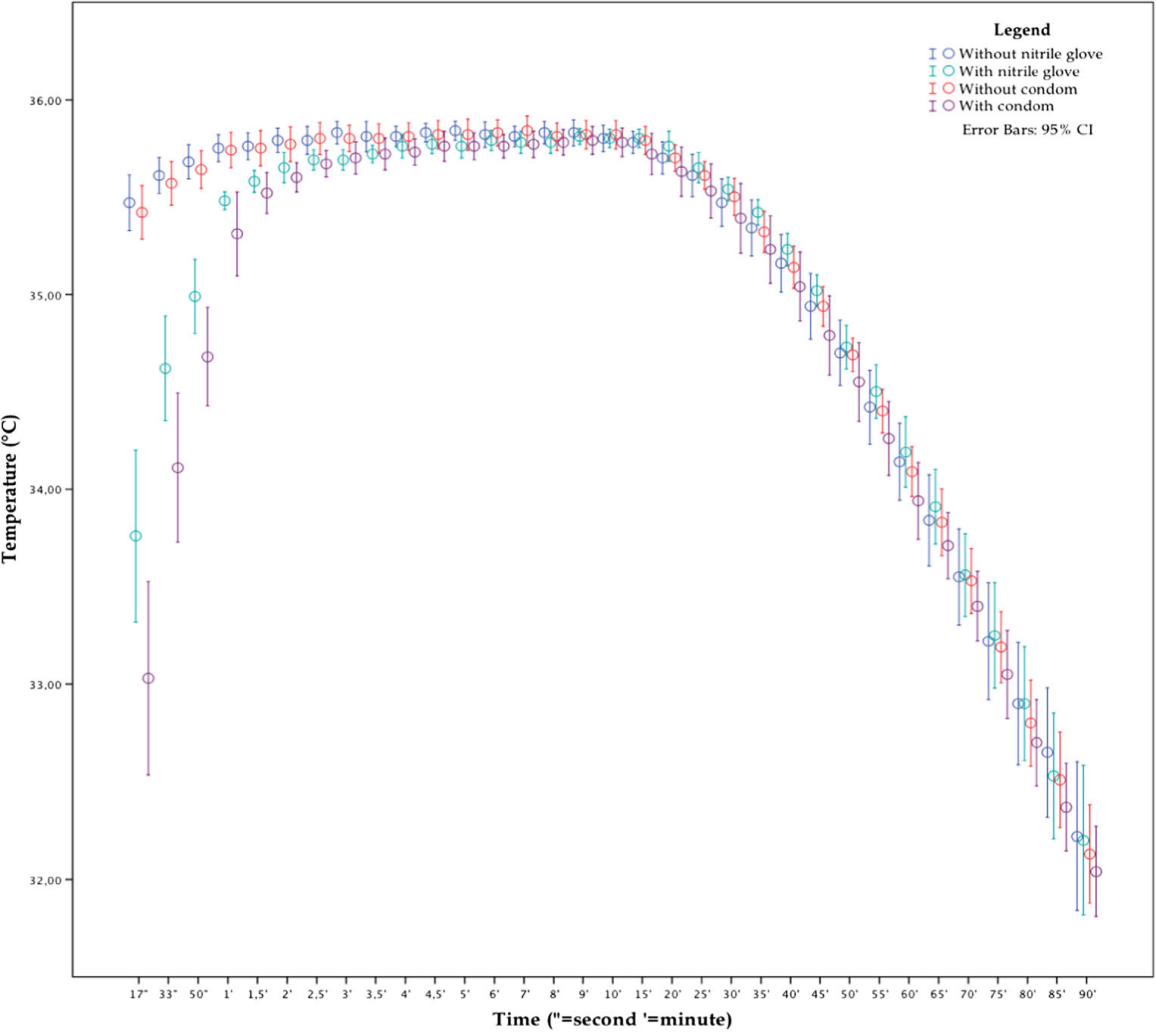
Temperature measurements from the experiment starting at 36 °C. The *blue* and *green dots* represent the measurements without and with a nitrile glove (*N* = 10). The *red* and *purple dots* represent the measurements without and with a condom (*N* = 10) (color figure online)

The results from the experiment with a starting temperature of 36 °C show a decreased precision, an enlarged 2σ standard deviation (95.4 % CI), during the first 2 min when comparing the group with condom versus the group without condom and the group with nitrile glove versus the group without nitrile glove (see Fig. 3). Further, the temperature measurements taken with an additional layer seem to lag behind the ones taken with the naked thermocouple for the first 4 min. The groups without an additional layer did not significantly differ from each other (*ρ* > 0.05).

The addition of a nitrile glove leads to a significant difference of the temperature measurements for the first 3.5 min and after 5 min, as can be seen in Table 2. The condom as an additional layer had a significant influence on the temperature measurement for the first 3 min (see Table 3). There was a significant difference between adding a nitrile glove and a condom for the first 50 s, and after 35 min until 1 h after insertion (see Table 4).

**Table 2:** Student’s *t* test on measurements taken after 17 s till 5 min, with a naked thermocouple compared with a nitrile glove covered thermocouple, at a starting temperature of 36 °C

Independent samples test — 36 °C — without nitrile glove versus with nitrile glove										
		Levene’s test for equality of variances		*t* Test for equality of means						
		*F*	Sig	*t*	*df*	Sig. (two-tailed)	Mean difference	Std. error difference	95 % confidence interval of the difference	
									Lower	Upper
17″	Equal variance assumed	6.121	0.024	8.3	18	0.000	1.71000	0.20507	1.27915	2.14085
	Equal variance not assumed			8.3	10.9	0.000	1.71000	0.20507	1.25801	2.16199
33″	Equal variance assumed	3.754	0.069	7.9	18	0.000	0.99000	0.12583	0.72564	1.25436
	Equal variance not assumed			7.9	11.1	0.000	0.99000	0.12583	0.71327	1.26673
50″	Equal variance assumed	4.862	0.041	7.5	18	0.000	0.69000	0.09220	0.49630	0.88370
	Equal variance not assumed			7.5	12.7	0.000	0.69000	0.09220	0.49037	0.88963
1′	Equal variance assumed	2.714	0.117	7.4	18	0.000	0.27000	0.03667	0.19297	0.34703
	Equal variance not assumed			7.4	15.5	0.000	0.27000	0.03667	0.19205	0.34795
1.5′	Equal variance assumed	0.658	0.428	4.6	18	0.000	0.18000	0.03944	0.09714	0.26286
	Equal variance not assumed			4.6	17.3	0.000	0.18000	0.03944	0.09690	0.26310
2′	Equal variance assumed	1.827	0.193	3.2	18	0.005	0.14000	0.04397	0.04762	0.23238
	Equal variance not assumed			3.2	17.3	0.005	0.14000	0.04397	0.04734	0.23266
2.5′	Equal variance assumed	0.512	0.484	2.6	18	0.020	0.10000	0.03916	0.01773	0.18227
	Equal variance not assumed			2.6	16.6	0.021	0.10000	0.03916	0.01723	0.18277
3′	Equal variance assumed	0.136	0.717	4.0	18	0.001	0.14000	0.03496	0.06655	0.21345
	Equal variance not assumed			4.0	17.8	0.001	0.14000	0.03496	0.06649	0.21351
3.5′	Equal variance assumed	4.669	0.044	2.2	18	0.038	0.09000	0.04014	0.00567	0.17433
	Equal variance not assumed			2.2	14.4	0.041	0.09000	0.04014	0.00411	0.17589
4′	Equal variance assumed	0.469	0.502	1.4	18	0.175	0.05000	0.03543	−0.02444	0.12444
	Equal variance not assumed			1.4	17.7	0.176	0.05000	0.03543	−0.02454	0.12454
4.5′	Equal variance assumed	0.000	1.000	2.0	18	0.062	0.06000	0.03018	−0.00342	0.12342
	Equal variance not assumed			2.0	18.0	0.062	0.06000	0.03018	−0.00342	0.12342
5′	Equal variance assumed	0.426	0.522	2.3	18	0.033	0.08000	0.03464	0.00722	0.15278
	Equal variance not assumed			2.3	17.4	0.033	0.08000	0.03464	0.00704	0.15296

**Table 3:** Student’s *t* test on measurements taken after 17 s till 4.5 min, with a naked thermocouple compared with a condom covered thermocouple, starting temperature of 36 °C

Independent samples test — 36 °C — without condom versus with condom										
		Levene’s test for equality of variances		*t* Test for equality of means						
		*F*	Sig	*t*	*df*	Sig. (two-tailed)	Mean difference	Std. error difference	95 % confidence interval of the difference	
									Lower	Upper
17″	Equal variance assumed	6.661	0.019	10.5	18	0.000	2.39000	0.22747	1.91209	2.86791
	Equal variance not assumed			10.5	10.4	0.000	2.39000	0.22747	1.88573	2.89427
33″	Equal variance assumed	4.474	0.049	8.29	18	0.000	1.46000	0.17607	1.09009	1.82991
	Equal variance not assumed			8.29	10.5	0.000	1.46000	0.17607	1.07039	1.84961
50″	Equal variance assumed	1.982	0.176	7.99	18	0.000	0.96000	0.12019	0.70750	1.21250
	Equal variance not assumed			7.99	11.5	0.000	0.96000	0.12019	0.69699	1.22301
1′	Equal variance assumed	0.971	0.338	4.18	18	0.001	0.43000	0.10290	0.21381	0.64619
	Equal variance not assumed			4.18	12.1	0.001	0.43000	0.10290	0.20601	0.65399
1.5′	Equal variance assumed	0.116	0.737	3.74	18	0.002	0.23000	0.06155	0.10068	0.35932
	Equal variance not assumed			3.74	17.6	0.002	0.23000	0.06155	0.10047	0.35932
2′	Equal variance assumed	0.316	0.581	3.29	18	0.004	0.17000	0.05175	0.06128	0.27872
	Equal variance not assumed			3.29	17.5	0.004	0.17000	0.05175	0.06106	0.27894
2.5′	Equal variance assumed	0.018	0.894	2.75	18	0.013	0.13000	0.04726	0.03071	0.22929
	Equal variance not assumed			2.75	17.3	0.013	0.13000	0.04726	0.03045	0.22955
3′	Equal variance assumed	1.200	0.288	2.12	18	0.048	0.10000	0.04714	0.00096	0.19904
	Equal variance not assumed			2.12	17.3	0.049	0.10000	0.04714	0.00068	0.19932
3.5′	Equal variance assumed	0.474	0.500	1.63	18	0.120	0.08000	0.04899	−0.02292	0.18292
	Equal variance not assumed			1.63	17.9	0.120	0.08000	0.04899	−0.02296	0.18296
4′	Equal variance assumed	0.445	0.513	1.84	18	0.082	0.08000	0.04346	−0.01131	0.17131
	Equal variance not assumed			1.84	18.0	0.082	0.08000	0.04346	−0.01132	0.17132
4.5′	Equal variance assumed	0.472	0.501	1.27	18	0.219	0.06000	0.04714	−0.03904	0.15904
	Equal variance not assumed			1.27	18.0	0.219	0.06000	0.04714	−0.03905	0.15905

**Table 4:** Student’s *t* test on measurements taken with a thermocouple covered with a nitrile glove compared with taken with a condom covered thermocouple, starting temperature of 36 °C

Independent samples test — 36 °C — with nitrile glove versus with condom										
		Levene’s test for equality of variances		*t* Test for equality of means						
		*F*	Sig	*t*	*df*	Sig. (two-tailed)	Mean difference	Std. error difference	95 % confidence interval of the difference	
									Lower	Upper
17″	Equal variance assumed	0.072	0.792	2.5	18	0.023	0.73000	0.29335	0.11369	1.34631
	Equal variance not assumed			2.5	18	0.023	0.73000	0.29335	0.11310	1.34690
33″	Equal variance assumed	0.632	0.437	2.5	18	0.024	0.51000	0.20669	0.07575	0.94425
	Equal variance not assumed			2.5	16	0.025	0.51000	0.20669	0.07221	0.94779
50″	Equal variance assumed	0.021	0.885	2.2	18	0.040	0.31000	0.14004	0.01579	0.60421
	Equal variance not assumed			2.2	17	0.041	0.31000	0.14004	0.01404	0.60596
1′	Equal variance assumed	2.401	0.139	1.8	18	0.096	0.17000	0.09690	−0.03357	0.37357
	Equal variance not assumed			1.8	9.8	0.111	0.17000	0.09690	−0.04650	0.38650
15′	Equal variance assumed	7.579	0.013	1.6	18	0.136	0.08000	0.05121	−0.02758	0.18758
	Equal variance not assumed			1.6	13	0.143	0.08000	0.05121	−0.03105	0.19105
20′	Equal variance assumed	2.817	0.111	2.0	18	0.062	0.13000	0.06540	−0.00741	0.26741
	Equal variance not assumed			2.0	15	0.066	0.13000	0.06540	−0.00952	0.26952
25′	Equal variance assumed	1.665	0.213	1.7	18	0.105	0.12000	0.07040	−0.02790	0.26790
	Equal variance not assumed			1.7	14	0.110	0.12000	0.07040	−0.03092	0.27092
30′	Equal variance assumed	3.561	0.075	1.8	18	0.091	0.15000	0.08386	−0.02619	0.32619
	Equal variance not assumed			1.8	11	0.101	0.15000	0.08386	−0.03459	0.33459
35′	Equal variance assumed	3.577	0.075	2.3	18	0.031	0.19000	0.08145	0.01889	0.36111
	Equal variance not assumed			2.3	12	0.039	0.19000	0.08145	0.01181	0.36819
40′	Equal variance assumed	3.157	0.092	2.2	18	0.040	0.19000	0.08596	0.00941	0.37059
	Equal variance not assumed			2.2	13	0.046	0.19000	0.08596	0.00402	0.37596
45′	Equal variance assumed	3.383	0.082	2.4	18	0.029	0.23000	0.09690	0.02643	0.43357
	Equal variance not assumed			2.4	12	0.036	0.23000	0.09690	0.01847	0.44153
55′	Equal variance assumed	1.241	0.280	2.3	18	0.032	0.24000	0.10349	0.02257	0.45743
	Equal variance not assumed			2.3	17	0.033	0.24000	0.10349	0.02121	0.45879
60′	Equal variance assumed	0.098	0.757	2.1	18	0.048	0.25000	0.11799	0.00211	0.49789
	Equal variance not assumed			2.1	18	0.048	0.25000	0.11799	0.00196	0.49804

### Starting temperature 30 °C

**Fig. 4:**
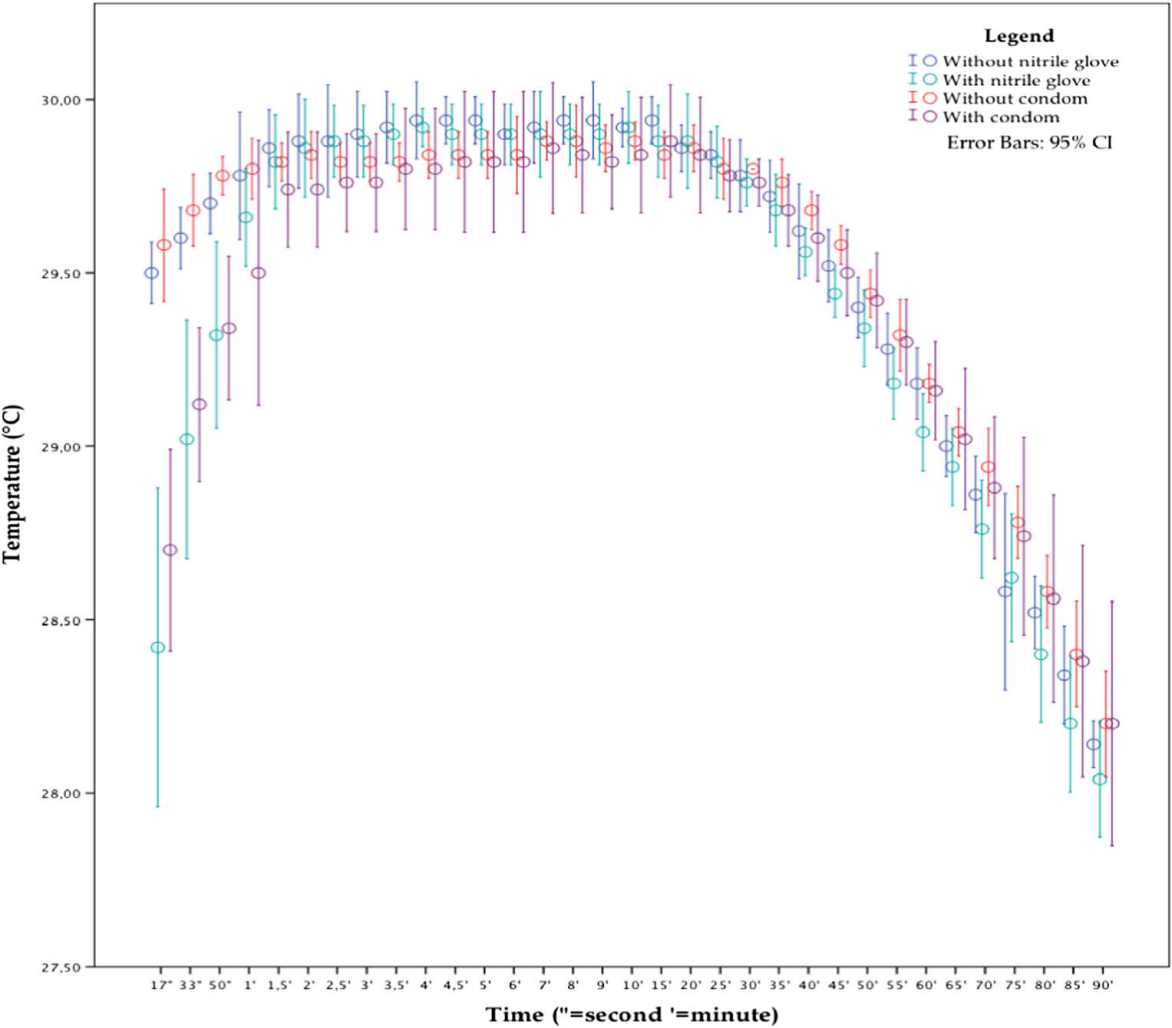
Temperature measurements from the experiment starting at 30 °C. The *green* and *blue dots* represent the measurements without and with a nitrile glove (*N* = 5). The *red* and *purple dots* represent the measurements without and with a condom (*N* = 5) (color figure online)

There appears to be a decreased precision for the first 2 min based on the results shown in Fig. 4. This decreased precision is also observed after 75 min at 28.5 °C. The temperature measurements taken with an additional layer seem to lag behind the ones taken with the naked thermocouple for the first 2 min, which is less than observed in the previous experiment with a starting temperature of 36 °C.

Adding a nitrile glove as an additional layer results in a significant difference during the first 50 s and also after 1 h (see Table 5). The condom as an additional layer also has a significant effect for the first 50 s (see Table 6). There was no significant difference between the two different additional layers (*ρ* > 0.05).

**Table 5:** Student’s *t* test on measurements taken after 17 s till 1 and 60 min, with a naked thermocouple compared with a nitrile glove covered thermocouple, at a starting temperature of 30 °C

Independent samples test — 30 °C — without nitrile glove versus with nitrile glove										
		Levene’s test for equality of variances		*t* Test for equality of means						
		*F*	Sig	*t*	*df*	Sig. (two-tailed)	Mean difference	Std. error difference	95 % confidence interval of the difference	
									Lower	Upper
17″	Equal variance assumed	10.75	0.011	6.41	8	0.000	1.08000	0.16852	0.69139	1.46861
	Equal variance not assumed			6.41	4.3	0.002	1.08000	0.16852	0.62438	1.53562
33″	Equal variance assumed	7.144	0.028	4.53	8	0.002	0.58000	0.12806	0.28469	0.87531
	Equal variance not assumed			4.53	4.5	0.008	0.58000	0.12806	0.23994	0.92006
50″	Equal variance assumed	2.246	0.172	3.73	8	0.006	0.38000	0.10198	0.14483	0.61517
	Equal variance not assumed			3.73	4.8	0.014	0.38000	0.10198	0.11525	0.64475
1′	Equal variance assumed	0.108	0.750	1.43	8	0.189	0.12000	0.08367	−0.07293	0.31293
	Equal variance not assumed			1.43	7.5	0.192	0.12000	0.08367	−0.07518	0.31518
60′	Equal variance assumed	0.094	0.767	2.56	8	0.034	0.14000	0.05477	−0.01369	0.26631
	Equal variance not assumed			2.56	8.0	0.034	0.14000	0.05477	−0.01360	0.26640

**Table 6:** Student’s *t* test on measurements taken after 17 s till 1 min, with a naked thermocouple compared with a condom covered thermocouple, starting temperature of 30 °C

Independent samples test — 30 °C — without condom versus with condom										
		Levene’s test for equality of variances		t Test for equality of means						
		*F*	Sig	*t*	*df*	Sig. (two-tailed)	Mean difference	Std. error difference	95 % confidence interval of the difference	
									Lower	Upper
17″	Equal variance assumed	5.434	0.048	7.3	8	0.000	0.88000	0.12000	0.60328	1.15672
	Equal variance not assumed			7.3	6.257	0.000	0.88000	0.12000	0.58927	1.17073
33″	Equal variance assumed	4.020	0.080	6.3	8	0.000	0.56000	0.08832	0.35634	0.76366
	Equal variance not assumed			6.3	5.670	0.001	0.56000	0.08832	0.34081	0.77919
50″	Equal variance assumed	5.592	0.046	5.7	8	0.000	0.44000	0.07746	0.26138	0.61862
	Equal variance not assumed			5.7	4.569	0.003	0.44000	0.07746	0.23509	0.64491
1′	Equal variance assumed	2.667	0.141	2.1	8	0.067	0.30000	0.14142	−0.02612	0.62612
	Equal variance not assumed			2.1	4.420	0.095	0.30000	0.14142	−0.07837	0.67837

### Starting temperature 27 °C

**Fig. 5:**
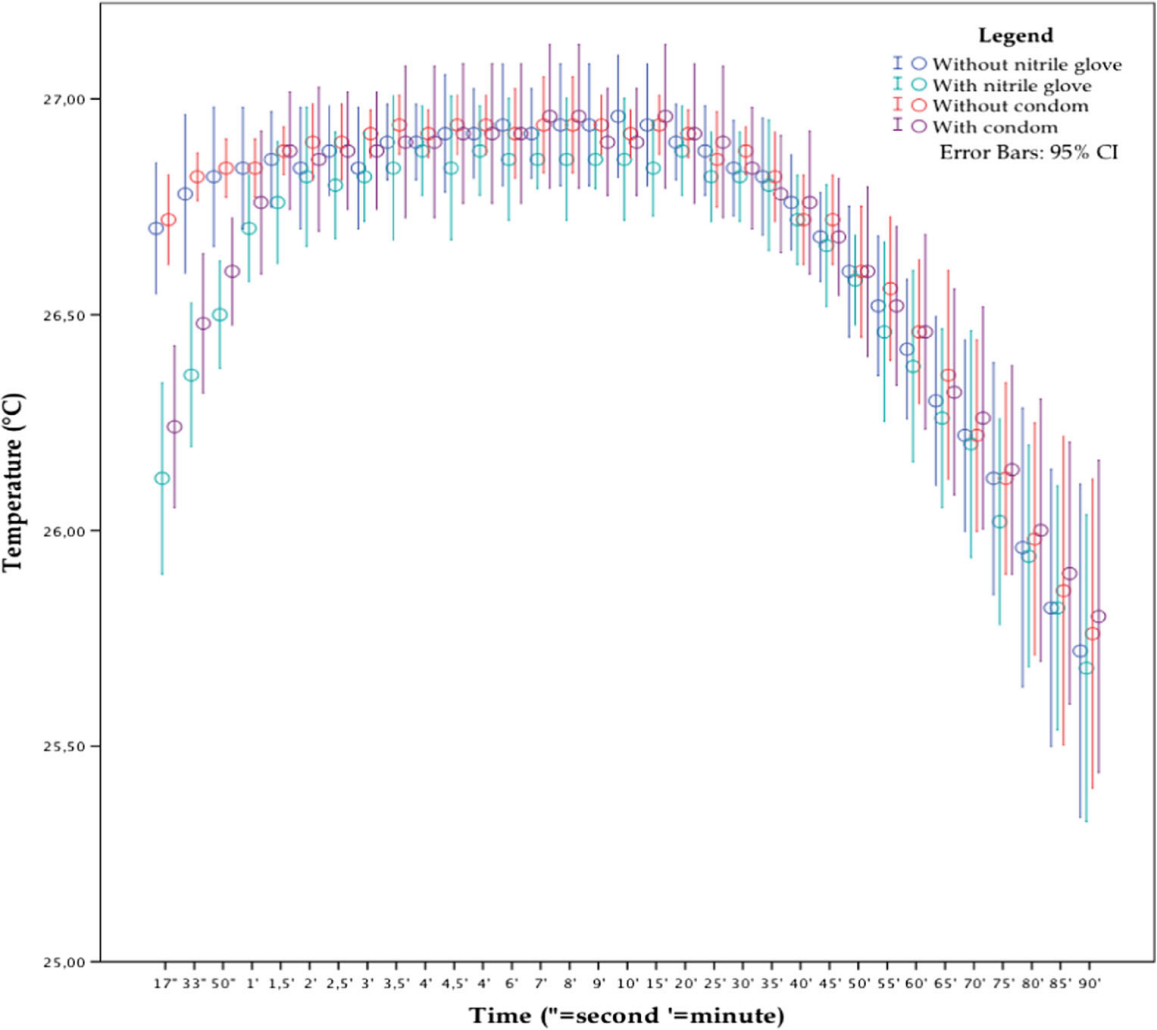
Temperature measurements from the experiment starting at 27 °C. The *green* and *blue dots* represent the measurements without and with a nitrile glove (*N* = 5). The *red* and *purple dots* represent the measurements without and with a condom (*N* = 5) (color figure online)

For the first minute and a half, the precision of the measurements with an additional layer is slightly decreased compared to the measurements without the extra layer. There also appears to be a delay in obtaining the right temperature of the body for the first 2 min (see Fig. 5). The observed phenomena are similar to the experiment with a starting temperature of 30 °C and are less than the experiment with a starting temperature of 36 °C. Compared with the measurements without an additional layer, the nitrile glove had a significant influence for the first 50 s (see Table 7). The condom also had a significant influence for the first 50 s (see Table 8). There was no significant difference between the group with a nitrile glove and a condom as an additional layer (*ρ* > 0.05).

**Table 7:** Student’s *t* test on measurements taken after 17 s till 1 min, with a naked thermocouple compared with a nitrile glove covered thermocouple, at a starting temperature of 27 °C

Independent samples test — 27 °C — without nitrile glove versus with nitrile glove										
		Levene’s test for equality of variances		*t* Test for equality of means						
		*F*	Sig.	*t*	*df*	Sig. (two-tailed)	Mean difference	Std. error difference	95 % confidence interval of the difference	
									Lower	Upper
17″	Equal variance assumed	1.57	0.246	6.0	8	0.000	0.58000	0.09695	0.35642	0.80358
	Equal variance not assumed			6.0	7.07	0.001	0.58000	0.09695	0.35123	0.80877
33″	Equal variance assumed	0.030	0.868	4.7	8	0.002	0.42000	0.08944	0.21374	0.62626
	Equal variance not assumed			4.7	7.92	0.002	0.42000	0.08944	0.21339	0.62661
50″	Equal variance assumed	0.526	0.489	4.4	8	0.002	0.32000	0.07348	0.15054	0.48946
	Equal variance not assumed			4.4	7.50	0.003	0.32000	0.07348	0.14854	0.49146
1′	Equal variance assumed	0.060	0.812	2.1	8	0.073	0.14000	0.06782	−0.01640	0.29640
	Equal variance not assumed			2.1	7.87	0.073	0.14000	0.06782	−0.01687	0.29687

**Table 8:** Student’s *t* test on measurements taken after 17 s till 1 min, with a naked thermocouple compared with a condom covered thermocouple, starting temperature of 27 °C

Independent samples test — 27 °C — without condom versus with condom										
		Levene’s test for equality of variances		*t* Test for equality of means						
		*F*	Sig.	*t*	*df*	Sig. (two-tailed)	Mean difference	Std. error difference	95 % confidence interval of the difference	
									Lower	Upper
17″	Equal variance assumed	1.252	0.296	6.2	8	0.000	0.48000	0.07746	0.30138	0.65862
	Equal variance not assumed			6.2	6.2	0.001	0.48000	0.07746	0.29213	0.66787
33″	Equal variance assumed	6.171	0.038	5.5	8	0.001	0.34000	0.06164	0.19785	0.48215
	Equal variance not assumed			5.5	4.9	0.003	0.34000	0.06164	0.18084	0.49916
50″	Equal variance assumed	2.415	0.159	4.7	8	0.002	0.24000	0.05099	0.12242	0.35758
	Equal variance not assumed			4.7	6.2	0.003	0.24000	0.05099	0.11621	0.36379
1′	Equal variance assumed	8.393	0.020	1.2	8	0.252	0.08000	0.06481	−0.06945	0.22945
	Equal variance not assumed			1.2	5.3	0.269	0.08000	0.06481	−0.08382	0.24382

## Discussion

### The model

The proposed model cannot be fully compared to the cooling of a human body, due to the difference in mass, the complexity of human tissue, the absence of postmortem biochemical processes, and the absence of a sphincter. Nonetheless, it is expected that the thermodynamic system is similar in its behavior because it strives toward the highest attainable entropy. Gel-based dummies have also been used in other cooling experiments, but so far, none have used a concentration that mimics the thermal conductivity of human muscle tissue [6, 8]. Therefore, an attempt was made by extrapolating the data from Zhang et al. to produce a model that is easily made for studying the cooling of a body [13]. Tissue substitute materials have also been developed for microwave and X-ray application; to our knowledge, there currently is no better substitute for objectively measuring the cooling of a body with such ease except using a euthanized animal [5]. Nonetheless, the observations are based on a model that was similar in mass and density in all experiments and performed under similar ambient circumstances (*ρ* > 0.05).

For the extrapolation of the concentration curve and associated formula, the data for 20 °C was used from Zhang et al. The highest temperature used in the experiments was 36 °C. Zhang et al. also reported the thermal conductivity for agar at 30 and 40 °C. Preference was given to the same concentration during all cooling experiments. According to the data from Zhang et al., the thermal conductivity changes when the temperature changes. Due to an increase in temperature, the thermal conductivity rises, thus at a temperature of 36 °C, the agar gel releases its energy more easily. The thermal conductivity of human tissue is also dependent on temperature, in a similar fashion as the agar solution. Only adipose, lung, and cancer tissue significantly differ according to the data from Valvano et al. [11].

### The results

At a higher starting temperature, 36 °C compared to 27 °C, it takes longer for the thermometer with an additional layer to obtain the right temperature. It is a natural phenomenon that with a larger temperature difference, it takes longer for the thermocouple to obtain the right value. This process is prolonged due to the obstructions between the two ambient temperatures. These layers have to adjust to the higher temperature, which results in loss of energy, and subsequently have to pass the remaining energy to the next layer. As stated earlier, the quasi-equilibrium will be achieved; it just takes longer.

During the experiments, two problematic factors were observed that further increase the delay and reduce the precision; air pockets between the added layer and the thermocouple and folding of the added layer. Nonmoving air has a very low thermal conductivity; hence, it has a good isolating property [12] (see Table 9 for an overview of different thermal conductivities).

**Table 9:** Different types of media with their associated thermal conductivity measured at 20–25 °C

Medium:	λ (W/(m °C))	Temperature (°C)	Reference
Spherical body of agar gel 3.6 % *w*/*v*	0.454	20	
Water	0.60	20	[3]
Air (21 % oxygen, nonmoving)	0.024	20	[12]
Nitrile, NBR	0.24	25	[4]
Rubber	0.14	25	[4]

The significant difference after 60 min between the group without the nitrile glove and with the nitrile glove at a starting temperature of 30 °C can be based on chance due to the relative low amount of measurements (*n* = 5). This significant difference has not been found at the higher starting temperature of 36 °C (*n* = 10).

## Conclusion

According to the guidelines of the Dutch Forensic Medical Society (Forensisch Medisch Genootschap), the rectal temperature should be taken by using a protective layer surrounding the thermocouple. The measurement should be taken within minutes after insertion and only when the temperature is stable. Although this seems to solve the issue of taking a false temperature reading, the best course of action is still to use a naked thermocouple based on the results of the carried out cooling experiments. The steepness of the curve is significantly decreased during the first 5 min, and the precision of the measurement is decreased, both due to the additional layer. There was a significant difference between using a nitrile glove versus using a condom as an additional layer, but both significantly differed from the naked thermometer. Thus, the minor improvement one can make by using the less influencing layer still does not justify the choice.
